# A data-driven global flood forecasting system for medium to large rivers

**DOI:** 10.1038/s41598-024-59145-w

**Published:** 2024-04-18

**Authors:** Wahid Palash, Ali S. Akanda, Shafiqul Islam

**Affiliations:** 1 Integrated Sustainability, Calgary, AB T2P 4H2 Canada; 2 Civil and Environmental Engineering, University of Rhode Island, Kingston, RI 02881 USA; 3 Civil and Environmental Engineering and The Fletcher School of Law and Diplomacy, Tufts University, Medford, MA 02155 USA

**Keywords:** Hydrology, Natural hazards

## Abstract

Losses from catastrophic floods are driving intense efforts to increase preparedness and improve response to disastrous flood events by providing early warnings. Yet accurate flood forecasting remains a challenge due to uncertainty in modeling, calibrating, and validating a useful early warning system. This paper presents the Requisitely Simple (ReqSim) flood forecasting system that includes key variables and processes of basin hydrology and atmospheric forcing in a data-driven modeling framework. The simplicity of the modeling structure and data requirements of the system allows for customization and implementation in any medium to large rain-fed river basin globally, provided there are water level or discharge measurements at the forecast locations. The proposed system's efficacy is demonstrated in this paper through providing useful forecasts for various river basins around the world. This include 3–10-day forecasts for the Ganges and Brahmaputra rivers in South Asia, 2–3-day forecast for the Amur and Yangtze rivers in East Asia, 5–10-day forecasts for the Niger, Congo and Zambezi rivers in West and Central Africa, 6–8-day forecasts for the Danube River in Europe, 2–5-day forecasts for the Parana River in South America, and 2–7-day forecasts for the Mississippi, Missouri, Ohio, and Arkansas rivers in the USA. The study also quantifies the effect of basin size, topography, hydrometeorology, and river flow controls on forecast accuracy and lead times. Results indicate that ReqSim's forecasts perform better in river systems with moderate slopes, high flow persistence, and less flow controls. The simple structure, minimal data requirements, ease of operation, and useful operational accuracy make ReqSim an attractive option for effective real-time flood forecasting in medium and large river basins worldwide.

## Introduction

Floods represent 39% of all natural disasters worldwide since 2000^1^, and they have gone up to 46% since 2010^2^. With changing climatic conditions, more and more floods are observed along with more extreme weather patterns. In addition, changes in land cover, land use, urbanization and infrastructure development in river basins are increasing flood risk and associated damage across the world. For instance, between 1960 and 2010, the number of recorded floods has increased by ten times^2^ and the economic losses have risen by seven-fold^3^. River flooding alone affects 21 million people and $96 billion in GDP worldwide each year, with the developing world increasingly exposed to flood risks more than the developed world^4^. If flooding conditions were better forecasted, then at-risk communities in flood-prone areas might have a chance to avoid the hazards of natural flooding becoming a disaster. Adequate preparation requires at least 5–10 days advance notice of a flood^5,6^. However, providing early flood warning through an operational flood forecasting system remains a key global challenge, particularly for resource-constrained regions of the world.

A global flood forecasting system is a network of data collection, modeling, and dissemination systems that are used to forecast and monitor the risk of flooding in different regions of the world. These systems typically use a combination of meteorological data, hydrological data, and geographic information, as described in the Global Flood Monitoring System (GFMS) by the University of Maryland^7^, to produce flood forecasts and alerts. These three types of data are used by computer models, such as the Global Flood Awareness System (GloFAS)^8^, to predict the likelihood and severity of flooding in different regions. Findings from these models are then used to develop alert systems, such as the Flood Forecasting and Early Warning System (FFEWS) by the World Meteorological Organization (WMO)^9^, to issue flood warnings to the public and emergency response agencies when a flood is imminent. A key challenge in the development and implementation of global-scale flood forecasting systems is the availability and access to data, complexity of modeling requirements, and ease of operationalization, as highlighted by various organizations such as the World Meteorological Organization (WMO) and the United Nations (UN), as well as in several academic journal publications^9,10^.

Over the last several decades, with an advanced understanding of atmospheric physics and river basin hydrology, deployment of upper atmospheric satellite observation networks, and increased computational power, our ability to integrate meteorological and hydrological modeling capabilities to develop regional and global-scale flood forecasting schemes has vastly improved^11–13^. A 2014 publication provided a detailed overview of as many as 14 global and four continental-scale flood forecasting schemes^13^. As of January 2023, however, most of these schemes are currently not in operation. In fact, currently there are only a few real-time flood forecasting systems available on a continental or global scale. To the best of our knowledge, only two global and four continental models exist for real-time flood forecasting^12^.

The Global Flood Awareness System (GloFAS) is one such global-scale scheme, jointly developed by the European Centre for Medium-Range Weather Forecasts (ECMWF) and the Joint Research Centre of the European Commission (EC). Since 2011, GloFAS has been providing ensemble streamflow forecasts at daily time steps and flood exceedance probabilities for large rivers around the world for up to 30 days in advance^8,12,14^. The scheme employs a distributed hydrological model at 0.1° spatial resolution with ensemble Numerical Weather Prediction (NWP) inputs covering the entire globe^14,15^. The other one is the Global Flood Forecasting and Information System (GLOFFIS) developed by Deltares, the Netherlands, and is based on a distributed hydrological model with precipitation inputs from several NWPs. It also provides ensemble streamflow forecasts for up to 16-day lead time at 0.5^0^ resolution and deterministic forecasts for 10-days at a 0.1^0^ resolution^12,16^.

There are several forecasting schemes that provide experimental forecasts for the short range (1–5-day). For example, the NASA-funded Global Flood Monitoring System (GFMS, http://flood.umd.edu/) provides forecasts for up to 5 days with the incorporation of satellite precipitation data as inputs to a hydrologic model covering a quasi-global grid (50°N–50°S)^17^. Similarly, the Floods Global system (http://floods.global) with the input of satellite precipitation as well predicts streamflow and flood exceedances in the next 3 days. Another is the Ensemble Framework for Flash Flood Forecasting (EF5) scheme (http://ef5.ou.edu/index.html/) that provides forecasts for up to 24 h in advance with the satellite precipitation inputs^18^.

An overview of current global and regional flood forecasting capabilities presented above reveals that there are relatively few real-time schemes. This is attributed to system complexity related to the numerous hydrometeorological, geophysical, and land use factors, as well as modeling complexity caused by uncertainties in precipitation data, lack of ground measurements, and scale mismatch in rainfall-runoff generation^19–26^. To address these challenges, we present a data-driven flood forecasting system that can be operationalized in low resource settings with relative ease, yet still provides quality forecasts at local, regional, and global scales. The proposed data-driven flood forecasting system, called the Requisitely Simple or ReqSim flood forecast system, includes a simpler modeling structure, utilizes readily available data, and is easier to implement in real time. ReqSim aims to learn from the data about how the river basin’s hydrologic system works by identifying and using adaptive relationships between a series of inputs and outputs^19^. Further details of the ReqSim system are available in the Methods section and have been previously documented in earlier publications^11,27^.

**Figure 1:**
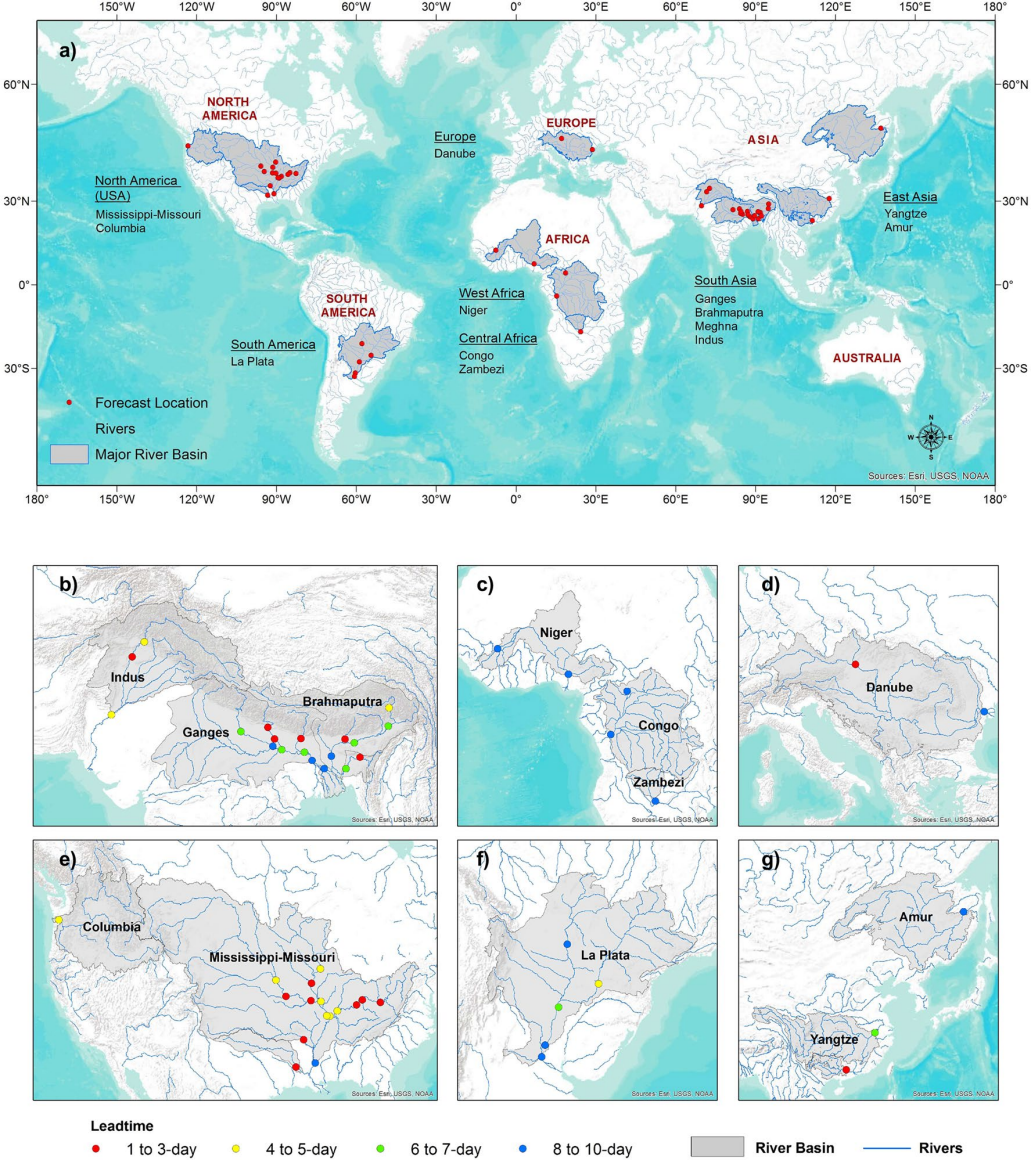
Flood forecasting by ReqSim for various regions of the world (**a**) and maximum lead time with reasonable forecasting accuracy (**b**–**g**).

The proposed system was applied to 51 watersheds in 13 major river basins from five continents (Fig. 1a). This paper presents the results of ReqSim application to the Ganges, Brahmaputra, Meghna (GBM) and Indus River basins in South Asia; the Niger, Congo and the Zambezi River basins in West and Central Africa; the Parana (La Plata) in South America; the Mississippi-Missouri in North America; and the Danube River basin in Europe (Fig. 1). The selection of forecast locations and watersheds was contingent upon the availability of recorded streamflow measurements, particularly post January 15, 2015, when the operational Global Forecast System (GFS) from the National Centers for Environmental Prediction (NCEP) started providing forecasted precipitation data. Observed river data and rainfall, along with the forecasted rainfall are crucial input data to the data-driven model presented here. However, for the La Plata River basin in South America and the Congo, Nigar, and Zambezi River basins in Africa, observed river data post January 15, 2015, were unavailable. Consequently, model development for these river basins relied on streamflow and rainfall data predating 2015, with observed rainfall considered as "true" forecast rainfall. For more detailed information on the methods and data employed, please refer to the Methods section at the end.

The ReqSim provides promising findings for short (3–5-day) to medium-range (6–10-day) flood forecasts for medium to large river basins. It has done so by combining available rainfall data from satellite and weather models, measurements of streamflow or water level, and day-to-day persistence of flow conditions. The daily rainfall averaged over a few large domains of a river basin (i.e., space-aggregated rainfall) and then further aggregated by the domains’ travel time (i.e., space–time aggregated) with a subsequent time lag adjustment is found to be a good predictor of basin’s rainfall-runoff response and downstream streamflow conditions. In essence, travel time for water from every domain to reach downstream forecast locations, space–time aggregated upstream domain rainfalls, and streamflow or water level measurements at forecasting locations are integrated into a regression model framework to forecast the likelihood of flooding at those locations. Using this approach, ReqSim is able to provide comparable or better forecasting accuracy relative to more complicated methods, such as detailed hydrologic modeling or satellite altimetry-based flood forecasting techniques^11,27^.

The paper begins by summarizing the performance of the ReqSim forecast in each river basin, and then examines factors that may impact the model's performance. These include river basin’s characteristics such as basin size, topography, hydrology, and degree of streamflow regulation in the river basin. Overall, the findings suggest that smaller river basins with flashier flood flow and shorter concentration time (travel time) have a shorter lead time for useful forecasts, while larger river basins with gradual rise and fall in flood and longer concentration time have longer lead times for useful flood forecasts^28^. To the best of our knowledge, this is perhaps one of the first studies to demonstrate the efficacy of flood forecasting over a large range of scales from different regions of the world.

### Findings from ReqSim

In this study, we have selected 51 watersheds in 13 major river basins from five continents to improve their operational flood forecasting capabilities with the application of the ReqSim system. The hydrology, meteorology, and geographic information of the selected river basins (and their sub-basins) in this study differ substantially. Unsurprisingly, ReqSim’s forecasting accuracy also differs significantly from basin to basin. Our goal with ReqSim is not to provide an off the shelf system that can be used anywhere but to provide a platform that can be customized with minimal time and resources for different basins with varied meteorological, hydrological, and geographic information. The following subsections present an overview of ReqSim’s streamflow forecast accuracy, including their strengths and limitations—for a range of river basins around the world. The results are divided into four basin scales based on their size: small (< 100,000 km^2^, in short < 100 K km^2^), medium (100 K to 500 K km^2^), large (500 K to 1000 K km^2^) and very large river basins (> 1000 K km^2^).

### ReqSim forecast accuracy

The section explores ReqSim’s forecast performance by presenting a range of quality forecast lead times alongside their corresponding performance metrics. This range of lead time demonstrates ReqSim’s capability to provide quality forecasts across different forecast locations and flood years. In this study, the ‘quality forecast’ is defined based on performance metrics such as coefficient of determination (R^2^), Nash Sutcliffe Efficiency (NSE) and Kling-Gupta Efficiency (KGE) values equal to or greater than 0.8, 0.7, and 0.8, respectively. The discussion of results follow these criteria throughout the paper. For more information on the evaluation criteria, please see the “Methods and data” section.

### South and East Asia

The evaluation of ReqSim performance demonstrates its ability to provide high-quality forecasts for various South Asian river basins, such as the Ganges (which drains Nepal, and part of India and Bangladesh) and the Brahmaputra (drains part of China, India, Bhutan, and Bangladesh), with lead times of 6–10 days (Fig. 1b).

The Coefficient of Determination (R^2^) and Nash–Sutcliffe Efficiency (NSE) values at all forecast locations along the Ganges River—from Elginbridge in Uttar Pradesh to Gangpur and Kanpur in Bihar, then Farakka in West Bengal, India to Hardinge Bridge in Bangladesh—have been 0.8 or greater during the 2017 and 2018 monsoon season (June–September). The R^2^ and NSE values over 0.8 suggest a quality flood forecast that can aid local authorities in making timely mitigation plans. The performance along the main Brahmaputra River on the eastern Tibetan Plateau in China is reasonably accurate with a 3-day lead time. The performance improves once the river flows through the Yarlung Tsangpo Grand Canyon and enters Arunachal Pradesh of India. For example, ReqSim can provide accurate forecasts 4–5 days advance at locations such as Dibrugarh near the China-India border to Guwahati in Assam, India, then 7 days ahead beyond the India-Bangladesh border at Bahadurabad inside Bangladesh (Fig. 1b), with R^2^, NSE values ranging from 0.6 to 0.75. The performance of the Brahmaputra River is highly encouraging compared to the Ganges, in which the steep terrain and high intensity rainfall results in significantly flashier flood flows, which are hard to predict beyond short-range (1–3 days)^29^.

In an earlier publication^12^, we presented a detailed comparison between ReqSim’s forecasts and several existing but more resource-intensive methods^30–32^ for these two river basins. Findings show that ReqSim was able to generate comparable, and in some cases better, flood forecasts across the flood season as well as during peak flood flow for 2007–2015. These results motivate us to explore the efficacy of Reqsim for other regions. It is also worthwhile to note that there is currently no operational flood forecasting scheme in India that provides forecasts beyond a 2–3-day lead time^28^.

The reason for better performance along the main rivers of the Ganges and Brahmaputra basin is attributed to several basin characteristics, such as basin size, topography, and rainfall patterns (Table 1). For example, forecast locations that show accurate forecast for a 7–10-day lead time typically have large upstream basin areas, an average slope of 3–6%, and average annual rainfall of about 1,400 mm in these two river basins. The ReqSim also provides quality forecasts at 4–7 days in advance for medium river basins with slope (annual rainfall) of 16–19% (1700 mm) and 2–5 days for small rivers with slope (annual rainfall) of 10–27% (2300 mm).

**Table 1:** ReqSim’s performance in the major river basins worldwide, presenting the maximum lead time for quality forecasts relative to basin scales, topography and hydrologic condition.

River basin (s)	Basin scale in size	No. of forecast locations	Area (in 1000 km^2^)	Slope (%)	Annual rainfall (mm)	Lead time (day)	NSE	KGE
Ganges–Brahmaputra	Small	7	< 100	10–27	2250	2–5	0.73–0.92	0.79–0.94
	Medium	3	100–500	16–19	1700	3–7	0.72–0.78	0.79–0.86
	Large	6	500–1000	3–6	1400	7–10	0.82–0.97	0.79–0.94
Indus	Small	Not available						
	Medium	2	100–500	20–23	810	2–3	0.86–0.87	0.92
	Large	1	500–1000	13–15	730	3–5	0.78	0.85
La Plata	Small to medium	N/A						
	Large	2	500–1000	1–1.3	1325	3–5	0.81–0.94	0.9–0.96
	Very large	3	> 1000	2–2.3	1400	5–10	0.81–0.97	0.80–0.95
Niger, Congo, and Zambezi	Small	Not available						
	Medium	2	100–500	1.2–1.5	1250	5–7	0.97–0.99	0.98
	Large to very large	3	> 500	1.1–1.8	1130	7–10	0.95–0.97	0.97
Mississippi	Small	Not available						
	Medium	6	100–500	0.9–3.0	1300	2–5	0.72–0.79	0.79–0.81
	Large to very large	4	> 500	2–2.5	1200	4–7	0.71–0.92	0.79–0.94
Missouri	Small	Not Available						
	Medium	2	< 1000	1.7	918	3–5	0.76	0.8
	Large to very large	3	> 1000	2.2–2.6	850	2–4	0.73–0.89	0.8–0.88

The ReqSim provides 3–4-day accurate forecasts for the Indus River downstream of the Guddu Barrage in central Pakistan, with R^2^ and NSE values above 0.8. The Guddu Barrage location is characterized by large basin areas with slope (annual rainfall) of 13–15% (730 mm) in the upstream region (Table 1). However, for medium-sized watersheds in this basin, the accuracy is somewhat limited with only 2 days forecasted for Khyber Pakhtunkhwa and Kalabagh Dam in Punjab, with upstream basin slope (annual rainfall) of 20–23% (810 mm). Despite this, the ReqSim's sub-basin scale forecasting for the Indus River is significant as it is the first flood forecasting scheme in this basin that allows for forecasts beyond a 1–2 day lead time^33^. The ReqSim therefore can play a crucial role in Pakistan by predicting the flow into major reservoirs and dams, and by enabling an efficient and timely dam operation strategy to reduce downstream flood risk.

The ReqSim has also shown high forecasting accuracy along the Yangtze River in China and the Amur River in China and Russia in the downstream areas, with a 3–7-day lead time (Fig. 1c) and R^2^ and NSE values ranging between 0.7 and 0.8.

**Figure 2:**
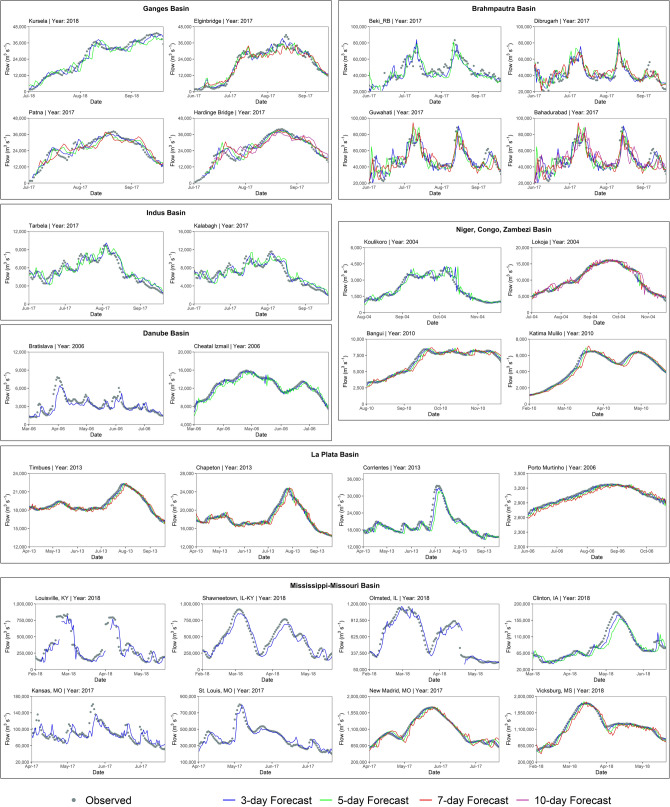
ReqSim forecast performance. The graph shows the comparison between observed and forecasted streamflow for 16 locations in major river basins around the world with different forecasting lead time.

Figure 2 presents a comparison between observed and forecasted streamflow at various lead times for multiple forecast locations in major river basins worldwide, including those in South Asia. The focus is on showing forecast performance at key locations within each basin, where the ReqSim model consistently provides high-quality forecasts over different lead times. However, not all stations are shown with the same lead time forecasts; instead, the figure presents performance up to the lead time where the model's accuracy meets the predetermined quality forecast criteria outlined earlier in this paper. This ensures that only forecast data meeting the established quality standards are included. Additionally, while there are several other locations within each river basin demonstrating quality forecasts, they were intentionally omitted to maintain figure readability. The periods in the figure vary across the river basins, primarily due to the availability of observed streamflow data.

### Western and Central Africa, and Central Europe

The ReqSim's forecasting accuracy in the Niger, Congo, and Zambezi River basins in Africa is very promising, particularly in medium to large river basins with upstream basin slope less than 2% and annual rainfall of 1200 mm (Fig. 1d and Table 1). The system provides accurate forecasts 3–7 days in advance for medium-sized watersheds upstream at two locations, Bangui on the Congo River in Central African Republic and Katima Mulilo on the Zambezi River in Zambia. On the Niger River in Nigeria and the Congo River in Congo, two other forecast sites, Lokoja and Kinshasa respectively, provide accurate flood forecasts up to 10 days in advance using the ReqSim. The R^2^ and NSE values for the lead time of quality forecasts range between 0.80 and 0.95 at all forecasting sites in these river basins. The Niger and Congo River basins have faced unprecedented floods in 2020, resulting in devastating human, social, and economic costs for the countries in the region^34^. These high forecasting accuracies for basins with different hydrological, meteorological, and geographic conditions suggest that the ReqSim has the potential to disseminate timely and accurate flood forecasts at longer lead times in these river basins in Africa, which is currently unavailable^35^. Figure 2 compares observed and forecasted streamflow at various lead times for multiple forecast locations in African and European basins considered in the study.

### South America

The ReqSim's performance in the La Plata River basin in South America, which drains parts of Brazil, Paraguay, Uruguay, and Argentina, has been limited to 2–5-day lead times. For example, ReqSim provides accurate forecasts up to 3 days in advance at Porto Murtinho on the Paraguay River and the Usina Itaipu Dam on the Parana River on the Brazil-Paraguay border. The R^2^ and NSE values for both these locations are above 0.8, with large basin areas upstream. However, beyond these forecast lead times, the model results are significantly influence by flow persistence, making them unusable for forecasting purposes. The slope of the basins varies from 1 to 1.3% with an annual rainfall of about 1325 mm. The performance improves after the confluence of the Paraguay and Parana Rivers, with increased basin area and flow persistence. For example, at the forecast locations of Corrientes, Chapeton, and Timbues on the Parana River in Argentina, ReqSim provides accurate forecasts up to 5 days in advance with R^2^ and NSE values well above 0.9. All of these forecasting sites have very large contributing areas upstream, with mild basin slopes between 2 and 2.3% and annual rainfall amounts over 1400 mm (Table 1).

### North America

The ReqSim's forecast performance in the Mississippi-Missouri River basin in the USA, North America's largest river basin, is limited to the Mississippi, Ohio and lower Missouri River only. In the Ohio River basin, ReqSim begins providing reasonable forecasts at least 2 days in advance from Louisville, Kentucky. The ReqSim modeling system continues to provide useful forecasts up to a 3-day lead time in the downstream, including the location of Olmsted, Illinois, close to the Ohio-Mississippi River confluence, with R^2^ and NSE values greater than 0.8. All of these forecasting sites have medium-sized river basins upstream with an average slope (annual rainfall) of 2.5–2.7% (1300 mm). We tested our forecast system at two more forecast sites upriver from Louisville, but found poor results, even though they had medium-sized watersheds upstream with a mild slope (2.8–3%). One of the reasons why forecasts are not useful beyond a 3-day lead time in the Ohio basin may be related to snowmelt-driven flood flows between February and April. The ReqSim works well when a near-linear relationship between space–time aggregated upstream rainfall and downstream streamflow is established. However, as the Ohio River basin has largely snowmelt-driven flood flows, it appears that the ReqSim is unable to provide accurate forecasts beyond a relatively short lead time in that basin. Therefore, we need to customize the ReqSim's modeling structure to address snow-driven flood forecasts.

The ReqSim begins by providing reasonably accurate forecasts up to 3 days in advance along the Mississippi River from the forecasting site at Clinton, Iowa and its downstream location Keokuk, also in Iowa. However, despite the significant increase in upstream watershed areas as a result of the Missouri River joining the Mississippi near St. Louis, Missouri, the performance of ReqSim does not improve until the Ohio River joins the Mississippi near Thebes in Illinois. The performance from Clinton to Thebes, therefore, remains consistent up to a 3-day lead time with both R^2^ and NSE values above 0.9. After the confluence of the Mississippi and Ohio Rivers, forecasts of up to 5–7 days in advance can be made for the locations of New Madrid in Missouri and Vicksburg in Mississippi, with an accuracy of R^2^ and NSE values more than 0.9.

The ReqSim is applied to several locations on the Missouri River, all of which have very large upstream basin areas with relatively mild basin slopes (2.2–2.6%) and low annual rainfall (800–900 mm). However, forecasts are not useful for these locations beyond a 2-day lead time. In the next section, we will discuss why ReqSim cannot provide accurate forecasts for longer lead times at some of the forecast sites in Missouri or other river basins, even though they have medium to large or very large watersheds upstream. These findings in Missouri are somewhat anomalous when compared to ReqSim's general forecasting performance, and will require further examination. On the other hand, the ReqSim forecast for the downstream of the Arkansas River at Murray Dam and the Columbia River at Port Westward in Oregon is encouraging for a 3-day lead time. The R^2^ and NSE values of forecasts at these locations range from 0.68 to 0.79. Figure 2 compares observed and forecasted streamflow at various lead times for multiple forecast locations in Mississippi and Ohio river basins.

### Relationships between basin scales and forecast lead time

**Figure 3:**
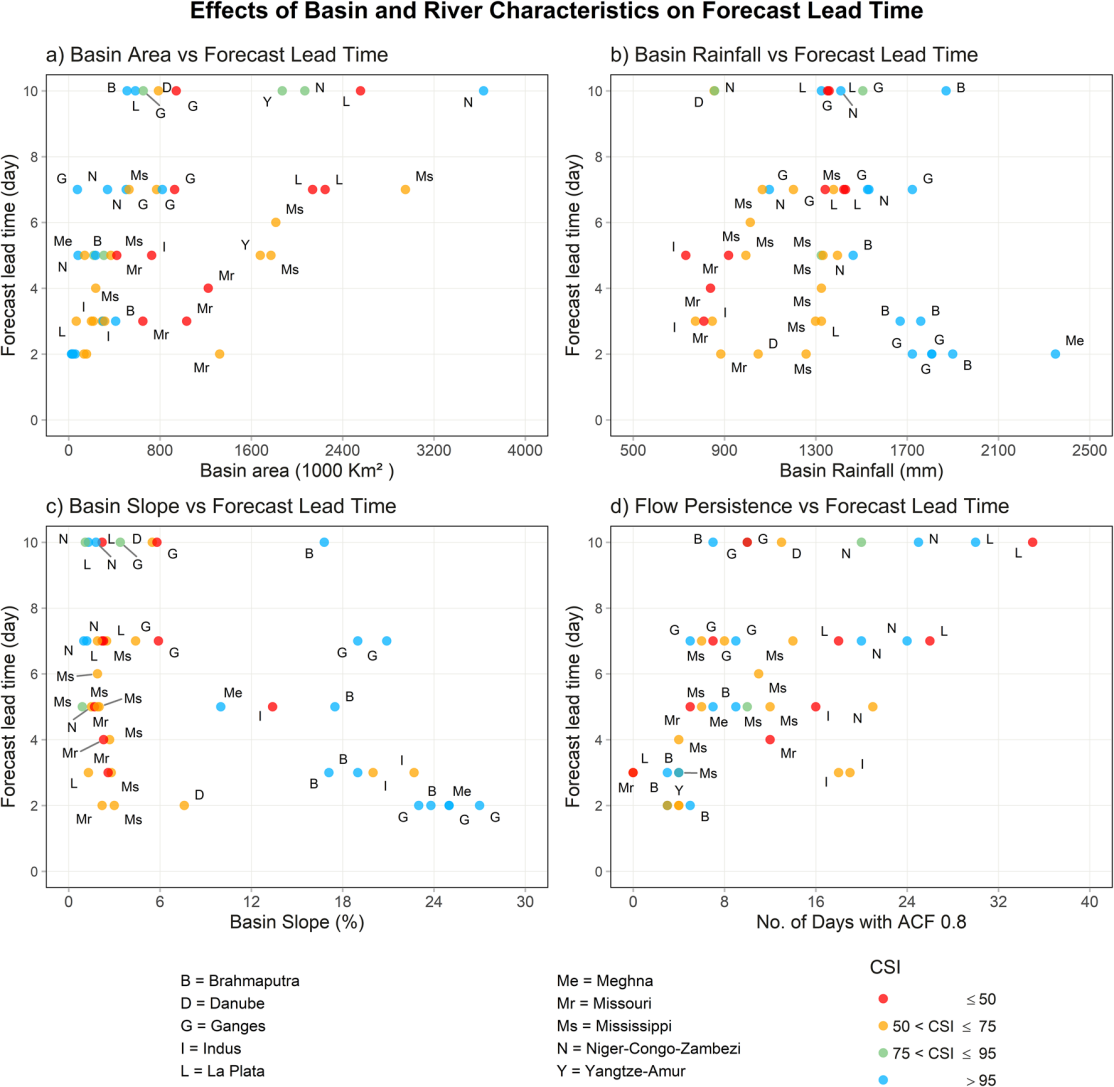
Variations of forecast lead time and accuracies for different basin scales and conditions. The figure shows relationships between river basin area and (**a**) basin topography (percent slope), (**b**) basin hydrometeorology (annual rainfall), (**c**) persistence in the streamflow (day-to-day correlation), (**d**) forecasting lead time (days).

A closer examination of the interrelationship between river basin scales, topography, hydrometeorology, streamflow persistence, and lead time (Fig. 3) reveals interesting insights into the role of basin scales and conditions related to flood forecasting accuracy.

For instance, generally, the lead time for quality forecasts tends to increase with larger upstream contributing basin areas (Fig. 3a) and with greater flow persistence (Fig. 3d), but it decreases as slope increases (Fig. 3c). This outcome is therefore not unexpected with the persistence-based model presented here. However, establishing a direct relationship with basin average annual rainfall (Fig. 3b) proves challenging when analyzing data from various river basins worldwide with diverse hydroclimatology. Nonetheless, patterns emerge within the same large basin or under similar hydroclimatic conditions. For instance, in the Ganges, Brahmaputra, and Meghna River basin (G, B, M points in Fig. 3b), the lead time for quality forecasts increases with a decrease in upstream basin rainfall. Conversely, in the Niger, Zambezi, and Congo River basins in Africa (N points in Fig. 3b), the Mississippi and Missouri River basins in North America (Ms and Mr points in Fig. 3b), and the La Plata River basin in South America (L points), the lead time for quality forecasts increases with an increase in basin rainfall. In other words, greater quality forecasts are available for the arid river basins than the semi-arid or dry basins.

Even considering these two contrasting features among these river basins with their respective basin slope and persistence, it becomes evident that higher basin rainfall in the Ganges, Brahmaputra, and the Meghna basin coincides with smaller watersheds in mountainous regions, therefore having higher average slope and lower flow persistence or runoff concentration time. On the other hand, lower rainfall or semi-arid regions in Africa, South and North America, considered in this study, are located in mountainous areas with greater slope, and thus less flow persistence. From this discussion, it is clear that topography, slope, and finally flow persistence affect flood forecast quality more profoundly, with longer persistence or time of concentration likely to provide higher forecast accuracy with longer lead time.

These relationships are neither linear nor easily generalizable; they vary significantly between medium to large (100 K to 1000 K km^2^) and for very large river basins (over 1000 K km^2^). Table 2 provides a summary of basin scales and conditions which may serve as a guide for other forecasting techniques like machine learning, satellite altimetry-based algorithms, or detailed physically based hydrological modeling applied to different basin sizes and hydrometeorological conditions.

**Table 2:** Role of basin area, topography and hydrometeorology on forecasting lead time and accuracy of ReqSim forecast.

Basin size	No. of forecast locations	Slope (%)	Annual rainfall (mm)	Lead time (day)
Small (< 100 K km^2^)	8	1.3–27 (19.5)	1300–4900 (2200)	2–5
Medium (100 to 500 K km^2^)	16	1–23 (8.5)	750–1750 (1250)	3–7
Large (500 to 1000 K km^2^)	12	1–19 (7)	700–1900 (1350)	3–10
Very Large (> 1000 K km^2^)	13	1–2.6 (2)	800–1400 (1100)	5–10

### The role of river connectivity on forecast lead time

There are, however, several contextual inconsistencies observed in our assessment of forecasting accuracy. For instance, there are several medium to very large watersheds, particularly in the La Plata and Missouri River basins, some of which do not have steep slopes or high annual rainfall and show enough persistence in their streamflow measurement in the downstream, yet the ReqSim failed to generate reasonably accurate forecasts at longer lead times (Fig. 3d,e). To explore this further, we examined the river connectivity from upstream to downstream, by using the connectivity status index (CSI)^36^. The lower the CSI value, the more the river is regulated along its path. As the ReqSim system is based on a near-linear relationship between upstream rainfall and downstream flow, any obstruction in the river's natural flow is likely to affect this relationship, which in turn reduces the likelihood of getting better forecasting accuracy for longer lead times.

**Figure 4:**
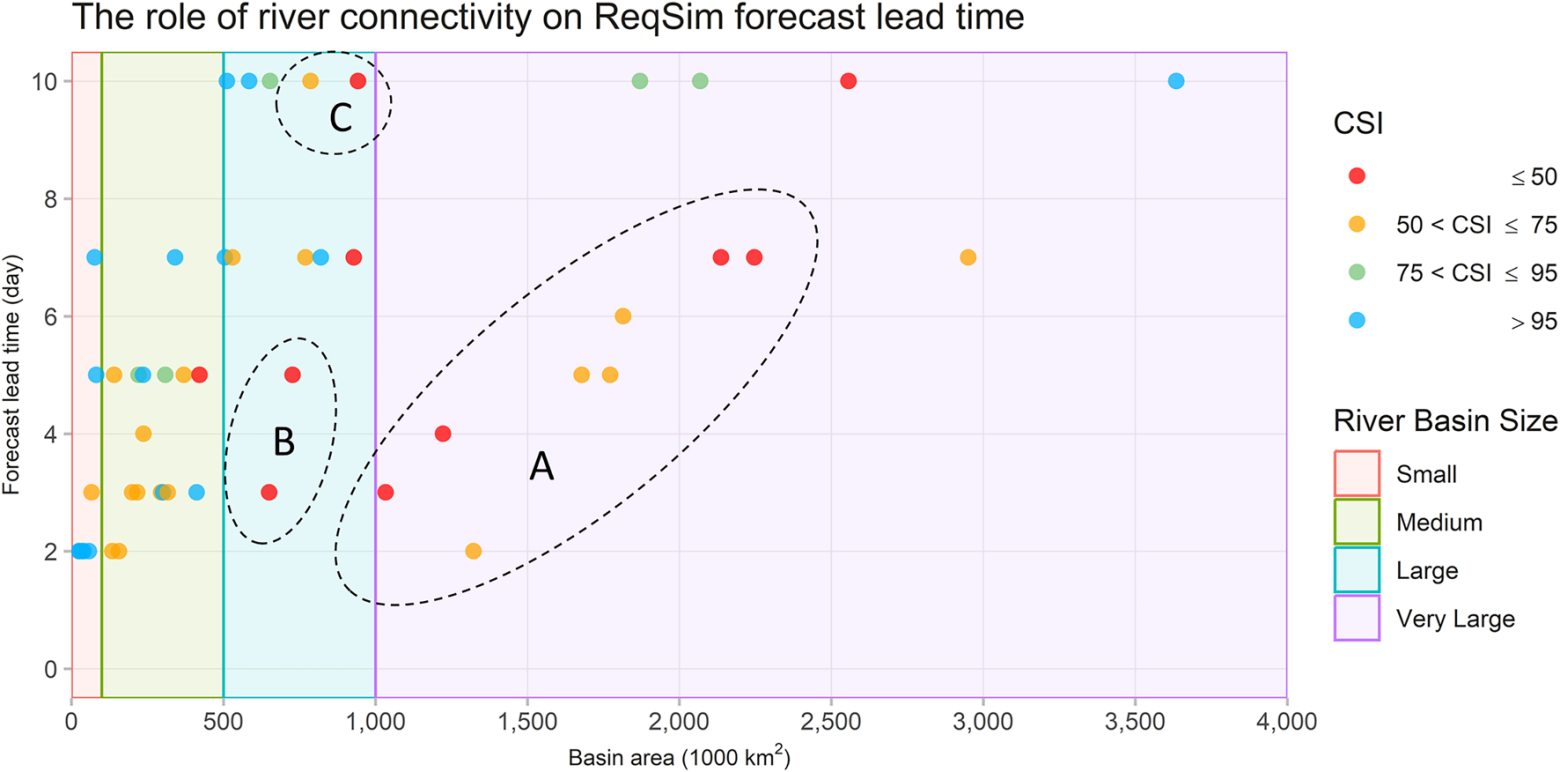
The role of river connectivity on ReqSim forecast lead time.

A recent global study^36^ has assessed the connectivity of 12 million km of rivers worldwide and reported that only 37% of rivers longer than 1000 km remain free-flowing over their entirety, while 23% of them flow uninterruptedly to the ocean. The study proposes a connectivity status index (CSI) to indicate the rivers' ability to flow naturally, while rivers with low CSI numbers are classified as regulated rivers. We analyzed the CSI values of rivers at each of our forecasting sites, compared them to the maximum lead time of ReqSim's forecasting accuracy, along with other characteristics of the basins such as basin size, slope, and hydrometeorology. Nearly all the rivers for which the ReqSim was unable to provide reasonably high forecasting accuracy for longer lead times fall within the CSI value of 75, which suggests that they are moderate to highly regulated rivers (See Zone A and B in Fig. 4). Therefore, the river connectivity and the extent to which the river is regulated will play an important role in customizing ReqSim for operational purposes for different basins.

The Zone C appears to be somewhat different than Zones A and B in Fig. 4. The ReqSim provides reasonably accurate forecasts for up to 10 days in advance for the Ganges River in Bihar State in India and further downstream in Bangladesh, even though the river is identified as highly regulated, with CSI values of around 50 or less. The Ganges River's CSI value, at locations such as Patna and Farakka in India, and Hardinge Bridge in Bangladesh, is around 50 mainly because of a very large barrage at Farakka that diverts the flow of the Ganges. However, the barrage is usually unregulated and left open during most of the flood season (June–September) in order to avoid flooding in upstream areas. As a result, the Farakka Barrage has relatively less impact on the river's natural flow during the flood season^37^ as the treaty is only meant for sharing the dry season flow, allowing for more accurate forecasting with longer lead time using ReqSim.

## Discussion

The state of the catchment and the predictability of hydrometeorological inputs are crucial factors that impact the accuracy of flood forecasting^11,27^. This study examines how the scale of a river basin affects the quality and lead time of flood forecasts by incorporating catchment characteristics (e.g., size and topography) and hydrometeorological conditions (e.g., average rainfall and streamflow persistence). Rainfall is a key input for physical process-based hydrological models and is commonly seen as a major source of uncertainty in flood forecasting^38–40^. Physical process-based models face challenges due to the mismatch between model equations and the variability of rainfall and runoff generation mechanisms, as well as uncertainties associated with parameter estimation, model calibration, and validation^11,12,17,19^. Yet there is a perception that increasing space–time resolution and physical parameterization can improve forecasting accuracy^27^. Such a perception may lead to the development of overly complicated models without significantly improving forecasting quality. In this paper, we present a data-driven flood forecasting system that strikes a balance between the complexity of model structure and the simplicity of operationalization. The proposed system is complex enough to capture key variables and rainfall-driven processes related to river basin hydrology, but simple enough in its structure, data requirements, and ease of operationalization for real-time flood forecasting. As we have shown, the ReqSim incorporates essential features of river basin hydrology such as rainfall, flow travel time, streamflow persistence, and flood propagation in its modeling structure, and can provide useful flood forecasts for medium to large river basins worldwide. It is important to note that the current version of ReqSim does not account for snowmelt-driven catchment hydrology. Consequently, the model is not suitable for river basins or watersheds where snowmelt-induced flood peaks are prominent.

Our application of ReqSim across the selected medium to large river basins on five continents revealed that factors like basin scale, topography, hydrometeorology, and the free-flowing nature of a river can have a significant impact on flood forecast performances. Overall, the ReqSim forecasts perform more effectively in river systems with moderate slopes, and semi-arid to wet hydroclimatic conditions. For instance, in the Ganges, Brahmaputra, and Meghna River basins in South Asia, ReqSim can provide 2–5-day accurate forecasts for small basins, 4–7 days for medium basins, and 7–10 days for large basins. However, in the Indus River basin, the forecast lead time is shorter at 1–2 days for medium basins and 3–4 days for large basins. Similarly, in African river basins such as the Niger, Congo, and Zambezi, ReqSim can provide accurate forecasts for medium basins within 3–5 days, and for large to very large basins within 5–10 days lead time. In the La Plata River basin in South America, ReqSim can provide accurate forecasts for large basins within 2–3 days, while it is up to 5 days for very large basins. In the Ohio River basins in the USA, ReqSim can provide accurate forecasts for medium basins up to 2–3 days in advance, while the same lead time is possible for large basins in the upper Mississippi basin. Additionally, ReqSim can generate 5–7-day accurate forecasts for the lower Mississippi River, which has a very large upstream basin area.

Our results further suggest that forecast accuracy and lead time are also impacted by human-caused interventions such as restrictions on natural flow in rivers, in addition to natural factors like basin size, topography, and hydrometeorological conditions. Rivers with more flow control measures, such as dams or barrages, are less likely to generate accurate forecasts with longer lead times. This highlights the importance of incorporating river connectivity information as well as operation strategy of river control structures in flood forecasting models, whether they are data-driven or physical process-based, in order to achieve accurate forecasts with longer lead times.

The ReqSim application has shown that large-scale weather systems captured by satellite and numerical weather models (e.g., GPM, TMPA, GFS, etc.) can be used in a data-driven model to obtain forecasts with reasonable accuracy without the need for complicated data processing operations. This is particularly useful in situations where access to measured data from upstream basins is limited, and other forecasting methods maybe resource-intensive and operationally prohibitive. Over the years, many complex and detailed hydrological and multi-modeling frameworks, as well as satellite altimetry-based flood forecasting schemes have been developed. However, some of these models have failed to provide expected results or are not currently operational. This is often due to the complexity of their model structure, the need for significant data and resources, and difficulties in transferring technology to local forecasting agencies. This is where the ReqSim system is novel and innovative in its ability to provide effective real-time flood forecasting on a global scale. Compared to other complex and resource-intensive methods, it provides a SMART alternative:

1. Simplicity (S): A simple structure makes the system easy to understand and operate, which is especially important in areas with limited resources or expertise. For example, in developing countries, where resources are limited and expertise in flood forecasting is not as developed, a simple system is more likely to be adopted and implemented effectively.
2. Minimal data requirements (M): The system requires minimal data inputs, making it more accessible to areas where data is scarce or difficult to collect. For example, in remote or rural areas, where weather and water level monitoring stations are not as prevalent, a system that requires minimal data inputs is more likely to be effective.
3. Affordability (A): A simple, minimal data requirement, easy-to-operate system is more cost-effective to implement and maintain than more complicated, resource-intensive methods. For example, a data-driven system that uses statistical or machine learning algorithms, rather than a more complex hydrological model, can be more cost-effective to develop and maintain.
4. Reliability (R): The system's accuracy is operationally useful, making it a reliable and valuable tool for making critical decisions related to flood management. It provides comparable forecasts to more complicated methods^12,13^.
5. Transferability and scalability (T): It can be easily scaled up or down depending on the area, making it more adaptable to different regions and contexts. For example, a data-driven system can be implemented in a limited number of critical forecasting locations and later be scaled up to cover a larger area.

Overall, a data-driven flood forecasting system is a SMART option for effective real-time flood forecasting across the globe. Such a cost-effective, adaptable, and easy to understand system will make it more accessible for disaster management and early warning in many areas around the world. We believe that these key features of the ReqSim system will make it widely applicable and actionable for medium to large river basins worldwide.

## Methods and data

### Model structure

Streamflow persistence (i.e., how the streamflow remains similar in a river over several days) and space–time aggregated daily rainfall for large upstream basin domains are good predictors of streamflow and floods in the downstream areas^11^. Using this as a guiding principle, our ReqSim flood forecast system consists of four components: (1) day-to-day persistence of measured streamflow or water level at the river point for which the flood forecasts are intended to generate, (2) spatially aggregated observed and forecasted daily rainfall for several upstream basin domains (i.e., domain-averaged daily rainfall), (3) temporally aggregated domain-averaged daily rainfall over the range of flow travel time duration (maximum and minimum no. of days) it takes for the water to travel from each domain to reach the forecast location downstream, and (4) flow travel time lag adjusted space–time aggregated domain rainfall. The model thus works by tracking key features of river basin hydrology, such as flow persistence, space–time aggregated rainfall, flow travel time, and the relationship of upstream rainfall to the downstream streamflow response at the forecast location. The model structure is as follows:

1 $$Q_{n} = \alpha_{n} Q_{t} + \beta_{n} Q_{t - 1} \sum\nolimits_{i = 1}^{m} {C_{i,n} R_{i,n} + \gamma_{n} }$$

where, ${Q}_{t+n}$ is the forecasted streamflow at $n$-day lead time; ${Q}_{t}$, and ${Q}_{t-1}$ are observed streamflow on forecast day $t$ and the day before that $t-1$, respectively; ${\alpha }_{n}$ and ${\beta }_{n}$ are model coefficients related to persistence$;$ and ${\gamma }_{n}$ is regression interception coefficient. ${R}_{i,n}$ are lagged space–time aggregated domain rainfall for a lead time of $n$ days, and ${C}_{i,n}$ are corresponding model coefficients for domain $i$ and lead time $n$.

2 $$R_{i,n} = \frac{1}{{T_{i,\max } - T_{i,\min } + 1}}\sum\nolimits_{{\tau = t - T_{i,\max } + n}}^{{t - T_{i,\min } + n}} {R_{i,\tau } }$$

${T}_{i,max}$ and ${T}_{i,min}$ are the maximum and minimum flow travel time from domain $i$ in no. of days; $t$ is the forecast day or 0-day. $\tau$ represents the time index, and ${R}_{i,\tau }$ is the daily rainfall of domain $i$ at time $\tau$.

**Figure 5:**
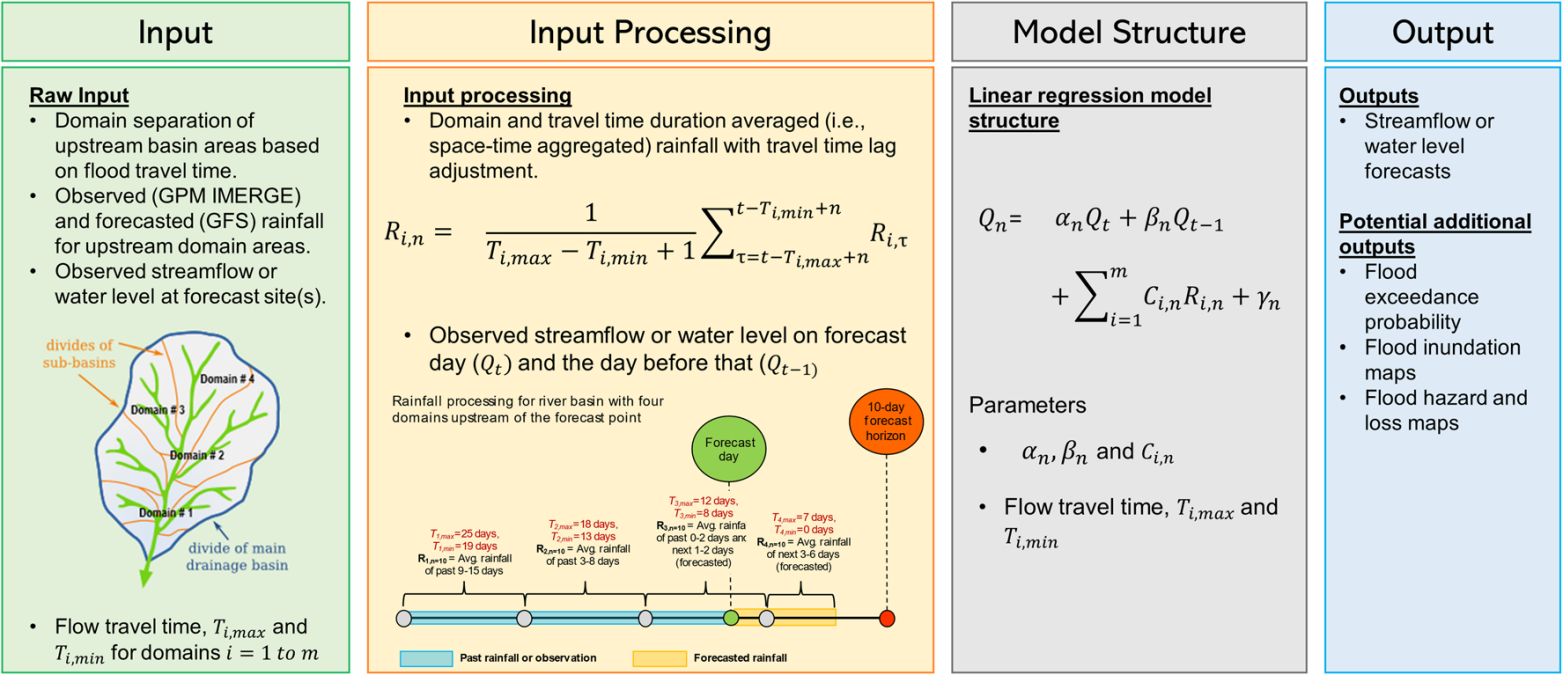
ReqSim flood forecast system’s model structure.

Figure 5 illustrates the ReqSim modeling system including the input processing approach and model structure. However, it is important to note that forecasted rainfall is considered for up to $$n$$-day lead time when viewed from the forecast day or 0-day. For instance, if the lead time $$n$$ is 10-day, $${T}_{i,max}$$ is 13 days and $${T}_{i,min}$$ is 6 days for a specific domain, the space–time aggregated domain rainfall $${R}_{i,n}$$ is calculated by averaging the daily domain rainfall from the past 3 days ($$t-{T}_{i,max }+n=0-13+10)$$ to forecasted rainfall for the next 4 days ($$t-{T}_{i,min }+n=0-6+10)$$. Furthermore, if $$t-{T}_{i,max }+n$$ and/or $$t-{T}_{i,min}+n$$ $$>t$$ in Eq. (2), then forecasted rain of $$k$$-day lead time is incorporated into the aggregation, provided that both $$t-{T}_{i,max }+n$$ and $$t-{T}_{i,min}+n$$ are less than or equal to $$(t+k$$). The value of $$k$$, which represent the lead time of forecasted rain, does not necessarily need to be equal to the lead time of flood forecasts, $$n$$, to generate skilled forecasts for medium to large river basins. It depends on factors such as basin size, topography, hydrology, and streamflow persistence. In an earlier publication^11^, we demonstrated that incorporating 6–7-day forecasted rain into the model generated useful 10-day streamflow forecast for the downstream Ganges River. However, for smaller and flashier rivers with less streamflow persistence, it may be advantageous to utilize a forecasted rain lead time closer to the lead time of flood forecasts.

Creating isochrones or flow travel time maps is a crucial step in developing the ReqSim system. We use the spatial hydrological analyst (SHA) in ArcGIS and the spatially distributed unit hydrograph (SDUH) concept^41^ to create these maps. The SHA uses the eight-direction pour-point algorithm to determine flow direction, accumulation, flow path and slope, then calculates the initial flow travel time using the mean velocity of the flow path derived from channel slop and roughness coefficient. The SDUH method, on the other hand, determines excess rainfall, establishes a time area histogram, and calculates the ordinates of the unit hydrograph, which is the incremental area divided by the representative time interval^42^. The SHA operation then updates the initial flow travel time from the SDUH's unit hydrograph generation, revises the flow velocity along the flow path, and finally calculates the flow travel time from each raster cell in the watershed to the basin outlet.

In the ReqSim, apart from regression coefficients ${\alpha }_{n}$, ${\beta }_{n}$, and ${C}_{i,n}$, maximum and minimum flow travel time or ${T}_{i,max}$ and ${T}_{i,min}$ are important parameters that influence the model results significantly. A detail overview of model structure, flow travel time calculations, and parameter sensitivity analyses are available in our earlier publication^11^. Developing a ReqSim model for an outlet of a watershed suggests that a linear regression model be developed incorporating the streamflow (or water level) measurements at that point and the observed and forecasted upstream rainfall.

### Data

We collected daily streamflow measurements, observed and forecasted rainfall of upstream contributing basin areas, river basins’ GIS files, land elevation, and land use-land cover data from multiple sources. Streamflow and rainfall are used in the linear model while the land elevation and land use data were used for preparing basin isochrones or flow travel time maps. A summary of data and their sources are presented in Table 3.

**Table 3:** Utilized data and their sources.

Data type	Source
Daily streamflow	(i) Global Runoff Data Centre (GRDC) (https://www.bafg.de/GRDC/EN/Home/homepage_node.html) (ii) Bangladesh Water Development Board (https://www.bwdb.gov.bd/) (iii) Institute of Water Modeling (IWM) (www.iwmbd.org) (iv) World Bank—South Asia Water Initiative (http://indiawbg.rap.ucar.edu/index.php/) (v) Pakistan Water and Power Development Authority (http://www.wapda.gov.pk/index.php/river-flow-in-pakistan) (vi) USGS National Water Information System (https://waterdata.usgs.gov/nwis/rt)
Tropical Rainfall Measuring Mission (TRMM) Multisatellite Precipitation Analysis (TMPA) 3B42 Real Time (RT) V07 (in short, TMPA 3B42RT V07) of 0.25^0^ special resolution	NASA Goddard Earth Sciences Data and Information Services Center (https://gpm.nasa.gov/data-access/downloads/trmm)
Global Precipitation Measurement (GPM) The Integrated Multi-satellitE Retrievals (IMERG) Late Precipitation L3 V06 (in short, GPM_3IMERGDL) of 0.1^0^ special resolution	NASA Goddard Earth Sciences Data and Information Services Center https://disc.gsfc.nasa.gov/datasets/GPM_3IMERGDL_06/summary?keywords=GPM_3IMERGDL_06
The National Centers for Environmental Prediction (NCEP) operational Global Forecast System (GFS) generated 1–10-day historical and real-time forecasted rainfall data of 0.25^0^ special resolution	Research Data Archive (RDA) https://rda.ucar.edu/datasets/ds084.1/
The river basin GIS shapefiles were collected	GRDC and USGS HydroSHEDS (https://hydrosheds.cr.usgs.gov/hydro.php)
Land elevation data of 90 m spatial resolution	Shuttle Radar Topography Mission (SRTM) generated Digital Elevation Model (DEM) (http://srtm.csi.cgiar.org/srtmdata/)
Land use data of 5′ spatial resolution	Global Mosaics of the standard (MODIS) land cover (MCD12Q1) (https://webmap.ornl.gov/wcsdown/dataset.jsp?dg_id=10004_31)

For the South Asian and USA basins, the collected streamflow data covers the period 2015–2018, where the first two years were considered as model calibration and the next two years as validation period. For the African, South American and East Asian river basins, the streamflow data were available prior to historical GFS forecasted rainfall data becoming available in 2015. Therefore, TRMM 3B42RT, which is observed rainfall data, was considered both as observed and forecasted data in the model for the period 2000–2010, where the first five years were considered as calibration and the next five years as validation period. Observed rainfall being used as forecasted rainfall is considered as using “perfect forecast” data, since the most accurate forecast data would behave just like an observed data. Nevertheless, the ReqSim model performance when using the “perfect forecasted” rainfall versus the “real forecasted” rainfall does not differ significantly, as we have shown in our earlier publication^11^.

### Forecast performance assessment

In this study, three distinct performance criteria were used to assess the forecast quality: the coefficient of determination (R^2^), Nash Sutcliffe Efficiency (NSE)^43^ and Kling-Gupta Efficiency (KGE)^44^. A forecast is considered useful and of high quality when these metrics meet or exceed certain threshold, specifically 0.8 for R^2^, 0.7 for NSE, and 0.8 for KGE.

3 $$R^{2} = 1 - \frac{{\sum\nolimits_{t = 1}^{N} {\left( {Q_{t,predict} - Q_{t,obs} } \right)^{2} } }}{{\sum\nolimits_{t = 1}^{N} {\left( {Q_{t,obs} - \overline{{Q_{t,obs} }} } \right)^{2} } }}$$

4 $$NSE = 1 - \frac{{\sum\nolimits_{t = 1}^{N} {\left( {Q_{t,obs} - Q_{t,sim} } \right)^{2} } }}{{\left( {\sum\nolimits_{t = 1}^{N} {Q_{t,obs} - \overline{{{\text{Q}}_{{\text{t,obs}}} }} } } \right)^{2} }}$$

5 $$KGE = 1 - (r - 1)^{2} + \left( {\frac{{\sigma_{sim} }}{{\sigma_{obs} }} - 1} \right)^{2} + \left( {\frac{{\mu_{sim} }}{{\mu_{obs} }} - 1} \right)^{2}$$

${Q}_{t,obs}$ is the observed series, ${Q}_{t,preict}$ is the predicted values of the observed series using a linear equation, and ${Q}_{t,sim}$ is the simulations. In the equation of KGE, $r$ is the correlation coefficient between observed and simulated data; ${\sigma }_{obs}$ and ${\mu }_{obs}$ denote the standard deviation and mean of observed series, respectively, while ${\sigma }_{sim}$ and ${\mu }_{sim}$ represent those of the simulated series. Generally, it is considered that model results with NSE values between 0.65 and 0.75 are good and anything above that is very good^45^. The KGE criterion^46^ is increasingly being used in place of NSE, with values between 0.7 and 0.82 considered average to slightly good model performance, while values above 0.82 are considered good to very good^47^.

By using the criteria explained above, we evaluated the performance of the ReqSim model at 51 forecast locations and identified the maximum forecast lead time for which the forecast quality is acceptable. Figure 3 shows these lead times in relation to the basin scale, such as small (< 100,000 km^2^ or < 100 K km^2^), medium (100 K to 500 K km^2^), large (500 K to 1000 K km^2^), and very large river basins (> 1000 K km^2^). Additionally, we examined how the basin's geophysical settings and hydrometeorological conditions affected forecast skill by analyzing the basin's annual rainfall (in mm) and slope (in percent), as well as streamflow persistence (in no. of days lag to reach auto-correlation function, ACF 0.8). We also considered another important factor, which is whether the river is free-flowing or regulated. The connectivity status index (CSI) of a river, as outlined in a 2019 published Nature article, provides a scale of the river's free-flowing or regulated character^36^. Only rivers with a high level of connectivity (i.e., less obstruction from control structures) are classified as free-flowing with CSI values over 95%. Conversely, rivers with low CSI numbers are regulated or controlled rivers. To determine the CSI of river reaches, the aforementioned study considered four types of river connectivity: longitudinal (connectivity between upstream and downstream), lateral (connectivity to floodplain and riparian areas), vertical (connectivity to groundwater and atmosphere), and temporal (connectivity based on seasonality of flows)^36^.

## Data Availability

The processed data that support the findings of this study are available from the corresponding author upon reasonable request.

## Code availability

The codes that support the findings of this study are available from the corresponding author upon reasonable request.

## References

[1] Debarati, G.S., Hoyois, P. & Below, R. *Annual Disaster Statistical Review 2016: The Numbers and Trends.* 79 (Brussels, CERD, 2016).

[2] IFRC. World disaster report 2020 (2020).

[3] Cullmann, J. *et al.* 2020 WMO state of climate services (2020).

[4] WRI. Aqueduct global flood risk country rankings | world resources institute (2015).

[5] WMO & Partnership, G. W. Flood forecasting and early warning (2013).

[6] Webster PJ, Hoyos C (2004). Prediction of monsoon rainfall and river discharge on 15–30-day time scales. Bull. Am. Meteorol. Soc..

[7] Wu H (2014). Real-time global flood estimation using satellite-based precipitation and a coupled land surface and routing model. Water Resour. Res..

[8] Alfieri L (2013). GloFAS—global ensemble streamflow forecasting and flood early warning. Hydrol. Earth Syst. Sci..

[9] WMO (2022).

[10] Perera D, Seidou O, Agnihotri J, Mehmood H, Rasmy M (2020). Challenges and technical advances in flood early warning systems (FEWSs). Flood Impact Mitig. Resil. Enhanc..

[11] Palash W (2018). A streamflow and water level forecasting model for the Ganges, Brahmaputra, and Meghna rivers with requisite simplicity. J. Hydrometeorol..

[12] Emerton RE (2016). Continental and global scale flood forecasting systems. Wiley Interdiscip. Rev..

[13] Bierkens MFP (2015). Hyper-resolution global hydrological modelling: What is next?. Hydrol. Process..

[14] Hirpa FA (2018). Calibration of the Global Flood Awareness System (GloFAS) using daily streamflow data. J. Hydrol..

[15] Alfieri L (2018). A global network for operational flood risk reduction. Environ. Sci. Policy.

[16] Werner M (2013). The Delft-FEWS flow forecasting system. Environ. Model. Softw..

[17] Wu H, Adler RF, Tian Y, Huffman GJ, Li H, Wang J (2014). Real-time global flood estimation using satellite-based precipitation and a coupled land surface and routing model. Water Resour. Res..

[18] Flamig ZL, Vergara H, Gourley JJ (2020). The ensemble framework for flash flood forecasting (EF5) v1.2: Description and case study. Geosci. Model Dev..

[19] Beven, K. *Rainfall-Runoff Modelling* (Wiley, 2012). 10.1002/9781119951001.

[20] Beven K (1989). Changing ideas in hydrology—The case of physically-based models. J. Hydrol..

[21] Clark MP, Hay LE (2004). Use of medium-range numerical weather prediction model output to produce forecasts of streamflow. J. Hydrometeor..

[22] Pappenberger F (2005). Cascading model uncertainty from medium range weather forecasts (10 days) through a rainfall-runoff model to flood inundation predictions within the European Flood Forecasting System (EFFS). Hydrol. Earth Syst. Sci..

[23] Charba JP, Samplatsky GF (2011). High-resolution GFS-based MOS quantitative precipitation forecasts on a 4-km grid. Mon. Weather Rev..

[24] Cloke HL, Pappenberger F (2009). Ensemble flood forecasting: A review. J. Hydrol..

[25] Dravitzki S, McGregor J (2011). Predictability of heavy precipitation in the Waikato River Basin of New Zealand. Mon. Weather Rev..

[26] Wood, E. F. *et al.* Hyperresolution global land surface modeling: Meeting a grand challenge for monitoring Earth’s terrestrial water. *Water Resour. Res.* **47**, (2011).

[27] Palash W, Akanda AS, Islam S (2020). The record 2017 flood in South Asia: State of prediction and performance of a data-driven requisitely simple forecast model. J. Hydrol..

[28] Priya, S., Young, W., Hopson, T. & Avasthi, A. Flood risk assessment and forecasting for the Ganges-Brahmaputra-Meghna river basins (2017).

[29] Bajracharya S (2015). Systematic evaluation of satellite-based rainfall products over the Brahmaputra basin for hydrological applications. Adv. Meteorol..

[30] Webster PJ (2010). Extended-range probabilistic forecasts of Ganges and Brahmaputra floods in Bangladesh. Bull. Am. Meteorol. Soc..

[31] Hossain F (2014). Proof of concept of an altimeter-based river forecasting system for transboundary flow inside Bangladesh. IEEE J. Sel. Top. Appl. Earth Observ. Remote Sens..

[32] Hossain F (2014). A promising radar altimetry satellite system for operational flood forecasting in flood-prone Bangladesh. IEEE Geosci. Remote. Sens. Mag..

[33] Shrestha MS (2019). Review of hydrometeorological monitoring and forecasting system for floods in the Indus basin in Pakistan.

[34] CARE. Niger flooding causes over 36,000 houses to collapse (2020).

[35] Thiemig V, de Roo A, Gadain H (2011). Current status on flood forecasting and early warning in Africa. Int. J. River Basin Manag..

[36] Grill G (2019). Mapping the world’s free-flowing rivers. Nature..

[37] Gain AK, Giupponi C (2014). Impact of the Farakka dam on thresholds of the hydrologic: Flow regime in the lower Ganges river basin (Bangladesh). Water (Switzerland)..

[38] Pappenberger F, Buizza R (2009). The skill of ECMWF precipitation and temperature predictions in the Danube basin as forcings of hydrological models. Weather. Forecast..

[39] Bauer P, Thorpe A, Brunet G (2015). The quiet revolution of numerical weather prediction. Nature.

[40] Wu H, Adler RF, Tian Y, Gu G, Huffman GJ (2017). Evaluation of quantitative precipitation estimations through hydrological modeling in IFloodS river basins. J. Hydrometeorol..

[41] Maidment, D. R. Developing a spatially distributed unit hydrograph by using GIS. In *Appl. geographic information systems hydrology water resources management. Proc. international conference, Vienna, 1993* (1993).

[42] Roy A, Thomas R (2007). Development of spatially distributed unit hydrograph for Bharathapuzha river basin. Int. J. Innov. Res. Sci. Eng. Technol. ISO..

[43] Moriasi DN (2007). Model evaluation guidelines for systematic quantification of accuracy in watershed simulations. Trans. ASABE.

[44] Gupta HV, Kling H, Yilmaz KK, Martinez GF (2009). Decomposition of the mean squared error and NSE performance criteria: Implications for improving hydrological modelling. J. Hydrol..

[45] Kitanidis PK, Bras RL (1980). Real-time forecasting with a conceptual hydrologic model: 2. applications and results. Water Resour. Res..

[46] Cheng KS, Lien YT, Wu YC, Su YF (2017). On the criteria of model performance evaluation for real-time flood forecasting. Stoch. Environ. Res. Risk Assess..

[47] Crochemore L (2015). Comparing expert judgement and numerical criteria for hydrograph evaluation. Hydrol. Sci. J..

